# Perspectives of healthcare professionals of the neuropsychiatric side effects associated with efavirenz and its management

**DOI:** 10.4102/hsag.v23i0.1076

**Published:** 2018-10-09

**Authors:** Razia Gaida, Ilse Truter, Christoffel Grobler

**Affiliations:** 1 Department of Pharmacy, Nelson Mandela University, South Africa; 2 Department of Psychology, Nelson Mandela University, South Africa; 3 Eastern Cape Department of Health, Bhisho, South Africa

## Abstract

**Background:**

Efavirenz is associated with neuropsychiatric side effects. The consequences of using efavirenz in human immunodeficiency virus (HIV)-positive patients with mental illness has not been conclusively established, the concern being that efavirenz may worsen the condition of an already mentally ill patient. The absence of guidelines and the lack of evidence for the use of efavirenz in this special population lead to uncertainty and, as a result, varying practices in the clinical setting

**Aim:**

To determine the experiences of healthcare professionals caring for mentally ill people living with HIV (PLWH) who are using efavirenz, the associated neuropsychiatric side effects and the management thereof.

**Setting:**

Eastern Cape, South Africa.

**Method:**

A qualitative, descriptive, exploratory design was used to understand the phenomenon under study and to share the experiences of the participants. Semi-structured interviews were conducted. The data were analysed using thematic framework analysis and coded by the researcher as well as an independent coder.

**Results:**

There were conflicting feelings concerning the use of efavirenz in PLWH with active mental illnesses. Some healthcare professionals were willing to prescribe and use efavirenz whilst others were not. All participants indicated that further elucidation in the guidelines on the possible side effects associated with efavirenz and suggested management strategies would be useful.

**Conclusion:**

The expansion of the South African National Guidelines for the Treatment of HIV should include descriptions of the side effects caused by antiretrovirals and management strategies thereof to empower healthcare professionals to make informed decisions regarding patient care for mentally ill PLWH.

## Introduction

Human immunodeficiency virus (HIV) is an infection that spreads through bodily fluids such as semen, blood, preseminal fluid, rectal fluid, vaginal fluids and breast milk (WHO 2016). The virus attacks the immune system of the host, rendering the host vulnerable to opportunistic infections resulting in the condition known as AIDS if left untreated (WHO 2016). Human immunodeficiency virus cannot be cured, but there are several classes of antiretrovirals (ARVs) used in the management of people living with HIV (PLWH) of which non-nucleoside reverse transcriptase inhibitors are one. Efavirenz together with nevirapine form part of this group of ARVs (Rossiter 2012:339). In 2008 in South Africa, the prevalence of HIV was 16.9% in people between the ages of 15 and 49 years (Freeman et al. 2008:490). In 2015 the overall HIV prevalence in South Africa was 11.2% translating to a total of 6.19 million persons living with HIV. For the same age group, 15–49 years, 16.6% were HIV-positive (Statistics SA 2015:1).

Literature strongly associates efavirenz with the incidence of neuropsychiatric side effects in HIV-positive patients (Gutierrez-Valencia et al. 2009:149; Kenedi & Goforth 2011:1803; Lochet et al. 2003:62). According to literature, at least 50% of patients initiated on efavirenz therapy will experience at least one neuropsychiatric side effect (Gutierrez-Valencia et al. 2009:149; Kenedi & Goforth 2011:1803; Lochet et al. 2003:62). These side effects usually occur within the first few weeks of treatment and will resolve spontaneously during that time (Blanch et al. 2001:339; Gutierrez-Valencia et al. 2009:155). Yet, it is possible for these neuropsychiatric side effects to start manifesting at a later stage in the patient’s treatment (Fumaz et al. 2005:563; Lochet et al. 2003:62, 63).

Three major risk factors for the development of neuropsychiatric side effects with the use of efavirenz are preexisting mental disorders, HIV disease progression and high plasma levels of efavirenz (Gazzard, Balkin & Hill 2010:68; Gutierrez-Valencia et al. 2009:155). Kagee et al. (2016:para. 4) aimed to determine the prevalence of preexisting mental disorders amongst South Africans seeking HIV testing. The most prevalent disorder amongst the 485 participants screened was major depressive disorder (14.2%) followed by a generalised anxiety disorder (5.0%) and post-traumatic stress disorder (4.9%). Alcohol-use disorder was found to be prevalent in 19.8% of the study population (Kagee et al. 2016:para. 12). Given that 43.9% of the study population screened positive for a mental disorder and that such patients are at a higher risk of experiencing neuropsychiatric side effects if prescribed efavirenz, it may be prudent to incorporate more vigorous mental health screening at the time of HIV testing to ensure that patients may receive the correct treatment both pharmaceutically and psychologically if tested positive.

Typically, HIV does not only affect patients biologically; there is also a psychological component. A review carried out in India (Srinivasan 2014:para. 4) stated that poor Indian families required extra assistance when caring for a sick family member. Assistance implies visits to the home from healthcare workers, food parcels and advice to the family for caring for the patient. In South Africa, some patients may be in similar situations and would benefit from such support. Human immunodeficiency virus also threatens other aspects of a patient’s life such as the way they perceive their life, goals and relationships (Srinivasan 2014:para. 4). A small study (*n* = 6) (Ramovha et al. 2012:4) performed at a public-sector psychiatric hospital in the Limpopo Province of South Africa found feelings of depression, hopelessness, anxiety and fear amongst the HIV-positive participants. Some expressed suicidal feelings and said that they did not feel that the staff maintained confidentiality concerning their HIV status particularly in the ward environment of the hospital (Ramovha et al. 2012:4).

In the general population as well as mental health users, HIV itself is able to cause neurocognitive decline by infecting the cerebral spinal fluid shortly after it enters its human host (Badkoobehi, Chana & Everall 2006:85). Viral activity inside the brain results in neuronal damage and, ultimately, cell death (Dube et al. 2005:238). Neurocognitive decline occurs subsequent to the infection and destruction of macrophages in the central nervous system (CNS) and the manifestation of such destruction can range from showing no symptoms to fully blown HIV-associated dementia depending on the extent of the neuronal damage (Antinori et al. 2007:1799; Dube et al. 2005:238). Efavirenz is able to suppress the viral load of HIV in the CNS, a desirable effect as the CNS serves as an important viral reservoir (Apostolova et al. 2015:para. 10; Tashima et al. 1999:864). However, because of the propensity of efavirenz to cause neuropsychiatric side effects, clinicians are hesitant to prescribe this agent to patients at risk of, or those who are already suffering from mental health issues.

There is no evidence regarding the consequences of using efavirenz in PLWH who are mental health users. The package insert (Adco-Efavirenz^®^ 2007) of efavirenz does not contraindicate the use of efavirenz in PLWH with an active psychiatric condition but rather warns against its use if a patient manifests severe psychiatric symptoms. The National Consolidated Guidelines in South Africa (2014:72, 77) states that efavirenz must not be used in patients with psychiatric illness and warns that it may cause persistent CNS-related side effects. The management strategy suggested is that efavirenz be substituted with nevirapine. If nevirapine cannot be tolerated, a boosted protease inhibitor such as the combination of lopinavir and ritonavir should be used instead (National Consolidated Guidelines for the Prevention of Mother-to-Child Transmission [PMTCT] of HIV and the Management of HIV in Children, Adolescents and Adults 2014:77).

Efavirenz has been shown to be virologically superior to both its potential substitutes (Nachega et al. 2008:6; Riddler et al. 2008:2103). Nevirapine carries a serious risk of hepatotoxicity as well as severe skin reactions (Schouten et al. 2010:4). Because of the inhibitory activity of lopinavir–ritonavir on enzymes in the CYP450 enzyme system, the plasma levels of drugs metabolised by the enzymes CYP3A4 and CYP2D6 will require dosage adjustments as a result of an increase in plasma levels (Cvetkovic & Goa 2003). These include drugs such as amitriptyline, buspirone, carbamazepine, paroxetine and risperidone, all of which are used in the treatment of psychiatric conditions (Ogu & Maxa 2000:422). The limitations of these agents make efavirenz the preferred choice.

The aim of the study was to explore the experiences of healthcare professionals caring for PLWH who are mental health users regarding the prescribing and use of efavirenz, the associated neuropsychiatric side effects and management thereof. The objectives were to understand whether healthcare professionals were aware of the possible side effects associated with efavirenz treatment, if mental health users were receiving efavirenz, if such patients were able to tolerate efavirenz and how any neuropsychiatric side effects were identified and how were they managed.

## Materials and methods

A qualitative, descriptive, exploratory design was used to understand the phenomenon under study and to share the experiences of the participants. The qualitative approach allows the researcher to test the validity of certain assumptions, claims or theories within a real-world context (Leedy & Ormrod 2005:134).

Criterion-based, non-probability, purposive sampling was used in selecting the study sample. Purposive sampling is concerned with providing a sample of information-rich participants (Struwig & Stead 2001:122). In order to participate in the study, participants had to be professional nurses working in the wellness centre of the public-sector clinics or a qualified general medical practitioner, registrar or psychiatrist working either in the clinics or at the public-sector psychiatric institutions which were included as study sites.

A total of 14 semi-structured interviews were conducted with consenting participants. This group consisted of five nurses and nine doctors. The participants recruited were involved in working with PLWH who are mental health users. Subsequent to ensuring the relevant permissions were obtained, the researcher telephonically contacted potential participants to enquire as to their interest in participating. If the response was positive, interviews were booked and conducted at the participant’s place of work. All interviews, except two, were conducted face-to-face. Two interviews were conducted telephonically as a result of logistic challenges. All the professional nurses interviewed were employed in the wellness centres of primary healthcare centres providing psychiatric services. All the medical officers interviewed were employed at specialised psychiatric hospitals.

The interview schedule was self-developed, keeping the aim of the study in mind, in order to explore the opinion of healthcare professionals on using efavirenz in patients who are mental health users through their lived experiences. The interview schedule was piloted with three participants to determine appropriateness and ease of understanding. A total of 10 semi-structured questions (see Table 1) were used to obtain relevant data. The questions focused on defining neuropsychiatric side effects associated with efavirenz and how these were managed. Any comments made by the participant during the interview that the researcher found compelling and pertinent were probed with follow-up questions. The interviews ended with asking for recommendations for changes that could be made concerning the National HIV/AIDS Guidelines in assisting healthcare professionals by providing clarity as to the use of efavirenz in PLWH who are mental health users and dealing with these side effects. The interviews were conducted in English because of it being a language the researcher and participants were able to fluently converse in. The interviews were recorded using a voice recorder and were later transcribed. The data were analysed using a thematic framework analysis. As reliability in qualitative studies is important, the researcher made a conscious effort to set aside preexisting conceptions and expectations when analysing the data.

**TABLE 1:** Interview schedule

Number	Questions
1.	Please would you state your qualification?
2.	How do you define a ‘neuropsychiatric side effect’?
3.	In your experience, approximately how long after efavirenz is initiated would neuropsychiatric side effects appear?
4.	Are the symptoms generally severe enough to warrant the discontinuation of efavirenz?
5.	With what symptoms, in your opinion, would a patient need to present in order for efavirenz to be discontinued?
6.	When a patient is diagnosed with severe efavirenz-induced neuropsychiatric symptoms, how would you manage the patient?
7.	Once neuropsychiatric symptoms have been stabilised or resolved, would you continue efavirenz treatment?
8.	How do you feel about prescribing efavirenz to a patient with a symptomatic mental illness?
9.	In your opinion, do patients who are switched to nevirapine because of an intolerable neuropsychiatric side effect of efavirenz experience side effect resolution?
10.	Do you feel that the national treatment guidelines for HIV and AIDS are sufficient in assisting the management of neuropsychiatric side effects? Where do you think improvements could be made? Please elaborate.

The data were coded by the researcher as well as an independent coder to ensure neutrality. The researcher familiarised herself with the data by listening to recordings of the interviews and transcribing the recorded interviews. The researcher noted any recurring ideas or themes in the interviews. Using these notes, the basis of the thematic framework was developed. This framework was flexible to allow the data to develop its own themes and sub-themes without the researcher forcing the data to fit certain expectations. Themes in each frame and supportive quotes were identified.

The final codes and themes presented in this paper had consensus between the coders. Literature was used to support the study findings. Every theme and sub-theme identified was substantiated by quotations from the raw data. This provided valuable data regarding the experiences of healthcare professionals with the use of efavirenz and the neuropsychiatric side effects associated with its use.

### Trustworthiness

Trustworthiness means achieving methodological soundness and adequacy. According to Guba’s model (in Krefting 1991:214) truth value, transferability, consistency and neutrality ensure trustworthiness in a qualitative study. Truth value in this study was achieved by spending sufficient time with participants to build a rapport before the interview, reflexivity with objectivity being maintained as far as possible throughout the research process, triangulation, peer examination and structural coherence (the experiences of the participants with efavirenz being prescribed to PLWH who are mental health users and the associated neuropsychiatric side effects and management thereof remained the main focus of the study). Transferability refers to the extent to which the findings can be applied to other groups, contexts or settings and its possibility was enhanced through purposive sampling and provided a dense description of the context, participants and findings. Consistency was achieved by the same strategies used to ensure trustworthiness, in addition to the co-coding procedure in which the data were coded by both the researcher and an independent coder. The concept of neutrality or the freedom of bias in the research procedure and results was ensured by triangulation and maintaining objectivity throughout the study. This means that to avoid the experiences being limited to one area, the participants interviewed were from different facilities. Additionally, the data collected from participants were verified as far as possible by the literature control and were analysed by both the researcher and an independent coder.

### Ethical considerations

Prior to commencement of the study, permissions were obtained from the Nelson Mandela Metropolitan University Human Research Ethics Committee (H14-HEA-PHA-001) and the Eastern Cape Department of Health as well as written consent from participants prior to participation.

## Results and discussion

The study sought to determine the experiences of healthcare professionals caring for PLWH who are mental health users prescribed and given efavirenz and its associated neuropsychiatric side effects and the management thereof. The following themes and sub-themes identified are outlined in Table 2 and elaborated on subsequently.

**TABLE 2:** Themes and sub-themes.

Theme	Sub-themes
**Theme 1** Knowledge of screening of patients with HIV for a history of psychiatric disorders prior to commencing efavirenz.	1.1 Effectiveness of screening for history of psychiatric disorders prior to initiating efavirenz.
**Theme 2** Participants described the side effects of efavirenz.	2.1 Most participants were aware of the possibility of the neuropsychiatric side effects of efavirenz. 2.2 Participants were unsure about the possibility neurological side effects of efavirenz.
**Theme 3** Use of efavirenz in patients with current psychiatric illness.	3.1 Participants comment on whether efavirenz may be used in patients with current psychiatric illness. 3.2 Identification and management of neuropsychiatric symptoms in patients with HIV taking efavirenz.
**Theme 4** Participants commented on whether the national treatment guidelines for HIV and AIDS are sufficient to assist practitioners in all aspects of disease management.	4.1 National treatment guidelines for HIV cover all possible aspects of treatment. 4.2 Participants provided possible improvements for the guidelines.

### Theme 1 – Effectiveness of screening for a history of psychiatric disorder prior to initiating efavirenz

A history of a psychiatric disorder increases the risk of developing neuropsychiatric side effects associated with efavirenz therapy (Schouten et al. 2010:789). Almost half of patients, without any history of a psychiatric disorder, initiated on efavirenz will experience neuropsychiatric side effects (Gutierrez-Valencia et al. 2009:149; Kenedi & Goforth 2011:1803; Lochet et al. 2003:62). The most commonly reported side effects include dizziness, insomnia, headache, abnormal dreams and impaired concentration (Arendt et al. 2007:148; Kenedi & Goforth 2011:1803; Nelson et al. 2011:337). These side effects occur most commonly in the first days of treatment and generally resolve within the first 4–6 weeks (Gutierrez-Valencia et al. 2009:155). This underlines the importance of baseline screening before the initiation of efavirenz. The screening for a history of psychiatric disorders is carried out by means of a single ‘yes’ or ‘no’ question on the record card before antiretroviral therapy (ART) is initiated (Gaida, Truter & Grobler 2016:para. 23). This is not sufficient for the screening of the mental health status of a patient. The healthcare staff working with PLWH could provide, at most, a vague definition of a neuropsychiatric side effect as pertaining to efavirenz. Whilst being able to give examples of side effects, the term itself could not be coherently explained. PN refers to a professional nurse and MO stands for medical officer:

‘…insomnia uhm, psychosis depression anything causing you know mood symptoms, mania uhm ya.’ (Interview 11, MO, female)

‘…a side effect that you can get from uh interaction of uh neurotransmitters from a circuit point of view and also the the receptors involved psychiatrically like the dopamine receptors and the noradrenaline and adrenaline receptors.’ (Interview 8, MO, male)

‘…a neuropsychiatric is when the nerve … the nerve of a psychiatric patient has been affected and that’s why the patient is having a neuropsychiatric side effect.’ (Interview 3, PN, female)

‘…they complain about eh bad dreams some dizziness ne headaches.’ (Interview 7, PN, female)

The side effects mentioned by both medical officers and professional nurses tended to be more psychiatric in nature than neurologic. As previously mentioned, the extent of screening for psychiatric disorders in the standardised medical record card for PLWH is single question asked to the patient on which they reply with either ‘yes’ or ‘no’. This may further negate the notion that mental health screening is important in PLWH. Of concern is that patients vulnerable to mental health disorders may be missed, could be commenced on efavirenz treatment and develop severe side effects resulting in referral to a psychiatric institution for further management. Thus, screening for psychiatric disorders amongst PLWH needs to be expanded. The National Comprehensive HIV Package of Care of Swaziland (2010:8, 10) recommend several follow-up questions to assess the mood of the patient and detect symptoms of depression.

### Theme 2. Participants described the side effects of efavirenz

#### Sub-theme 2.1 Participants had diverse knowledge regarding the possibility of neurological side effects of efavirenz

The commonly reported side effects caused by efavirenz such as dizziness, headache, insomnia, abnormal dreams and impaired concentration include both neurological and psychiatric symptoms. The side effects reported by participants varied in nature; some quoted only psychiatric side effects and others a combination:

‘…the reaction you got after you took maybe a medication which induces you know those side effects of neurological it’s like it could be related to your mentality would be disturbed…’ (Interview 4, PN, male)

‘…they will be having that dizziness … that blurred vision at night then what is also going to happen they will others they will have they will see some things that they are afraid of [*visual hallucinations*]…’ (Interview 6, PN, female)

‘…they complain about eh bad dreams some dizziness ne headaches.’ (Interview 7, PN, female)

‘…can expect um psychosis some patients can become psychotic after taking efavirenz.’ (Interview 8, MO, male)

Considering that the above responses were provided by qualified professional nurses or doctors, the vocabulary used when explaining various medical phenomena was non-scientific.

Although simple terms may be used with patients, healthcare professionals need to be clear on the concept they are explaining. Neither medical officers nor professional nurses could effectively describe the side effects in scientific nor lay language.

### Theme 3. Use of efavirenz in patients with current psychiatric illness

#### Sub-theme 3.1. Participants comment on whether efavirenz may be used in patients with current psychiatric illness

The participants were asked if they would prescribe efavirenz to a patient with an active mental illness. Efavirenz is not specifically contraindicated in mental health users (Adco-Efavirenz^®^ 2007). The National Consolidated Guidelines (2014:77) in South Africa warn that efavirenz may cause persistent CNS toxicity such as abnormal dreams, depression or mental confusion. The risk factors for these side effects are cited as depression or other mental disorder either previous or at baseline (National Consolidated Guidelines for PMTCT and the Management of HIV in Children, Adolescents and Adults 2014:77):

‘…if they can tolerate it … no problem prescribing … efavirenz to them.’ (Interview 11, MO, female)

‘…somebody who’s got risk like to have a mental illness … for such a patient … I wouldn’t want to put on on efavirenz because that patient is already predisposed you know.’ (Interview 13, MO, male)

‘We don’t we don’t we don’t do it.’ (Interview 6, PN, female)

‘I don’t recommend it.’ (Interview 9, MO, male)

‘…no one can really say whether efavirenz is actually contraindicated … people really just do what they want…’ (Interview 11, MO, female)

‘I don’t have an idea really we don’t initiate actually the patient come from the HIV clinics here.’ (Interview 9, MO, male)

The responses were varied as some said ‘yes’ and others ‘no’. The conclusion from the literature is there is no solid evidence for either decision. ART is initiated by nursing sisters in primary healthcare facilities in South Africa; therefore, clarity in guidelines as well as comprehensive screening tools are essential for ensuring that patients receive appropriate treatment. Interestingly, the idea was expressed that efavirenz, when used as part of the fixed dose combination (FDC), induced fewer neuropsychiatric side effects as compared to when efavirenz is used as an individual agent:

‘FDC here that contains efavirenz and we don’t really, you know, see many um you know efavirenz-related psychiatric symptoms.’ (Interview 1, MO, female)

‘…most patients that I have seen they’ll present it [*neuropsychiatric side effects*] in the six months of initiation of efavirenz. But but that is uh that applies to when efavirenz is used as a single drug, not as part of the uh combination fixed dose combination.’ (Interview 13, MO, male)

#### Sub-theme 3.2. Identification and management of neuropsychiatric symptoms in patients with HIV taking efavirenz

The participants were asked at what point would they decide to discontinue efavirenz therapy.

‘…impair the patient’s functioning, you know.’ (Interview 13, MO, male)

‘…influencing their function or affecting their function then I would stop.’ (Interview 11, MO, female)

‘…we have to involve the uh um the patient uh doctors who managed the HIV you must refer back to them.…’ (Interview 9, MO, male)

‘…normally we don’t fiddle with that we just treat them psychiatrically.’ (Interview 9, MO, male)

It would appear as though clinicians in specialist facilities are unwilling or unable to treat patients holistically and will treat only the condition they consider their speciality. They expressed the opinion that the person who initiated the ART should remain responsible for the HIV treatment of the patient. This results in clinicians not being familiar with HIV in practice and having, at best, a theoretical knowledge of the condition:

‘I don’t have an idea really we don’t initiate actually the patient come from the HIV clinics here.’ (Interview 9, MO, male)

‘I’m not actually…ya knowledgeable (laughs) I must go and read up.’ (Interview 11, MO, female)

‘…wouldn’t be confident but I don’t have much information.’ (Interview 13, MO, male)

‘But I must say, I have very limited experience….’ (Interview 14, MO, female)

Patients presenting with acute psychosis are managed using antipsychotic agents:

‘…appropriate antipsychotics trying to get the patient, you know, psychiatrically stable again.’ (Interview 1, MO, female)

‘…often you have to put the patient on a low dose of an antipsychotic.’ (Interview 13, MO, male)

The neuropsychiatric side effects associated with efavirenz may be treated according to their severity (Turjanski & Lloyd 2005:60). Mild symptoms can be monitored and treated pharmacologically if necessary, whereas more severe symptoms may warrant the discontinuation of efavirenz. Healthcare workers demonstrated an awareness of the more severe side effects that significantly impaired the quality of life of the patient. The suggested management of these side effects by participants was to discontinue efavirenz treatment and replace it with a viable alternative. Jonsson and Joska (2009:23) indicate that it is safe to use antipsychotic agents in PLWH who present with acute psychosis. These agents should always be used at the lowest possible dose for the shortest duration possible.

### Theme 4. Participants commented on whether the national treatment guidelines for HIV and AIDS are sufficient to assist practitioners in all aspects of disease management

#### Sub-theme 4.1. National treatment guidelines cover all possible aspects of treatment

Whilst the National Guidelines (2014:76–78) focus on some adverse effects of ARVs, the information is superficial. Certain conditions such as fat redistribution syndrome, bone density reduction and renal tubular dysfunction caused by tenofovir and abacavir hypersensitivity reaction are discussed in more detail. As most PLWH are diagnosed and initiated on treatment in primary healthcare facilities, the staff, particularly nurses, in these facilities view the guidelines as a definitive document. Therefore, further elucidation on the major side effects caused by each agent and management strategies thereof would prove useful:

‘I think there is room for improvement.’ (Interview 1, MO, female)

‘…think improvements need to be done….’ (Interview 4, PN, male)

‘…more information is still needed there.’ (Interview 5, PN, female)

Clarity concerning the identification and management of neuropsychiatric side effects would give healthcare staff more confidence when faced with such challenges:

‘…bit scared when dealing with a neuropsychiatric [*side effect*].…’ (Interview 12, MO, female)

#### Sub-theme 4.2. Participants expressed a desire for expansion of guidelines

The current guidelines available in South Africa for the treatment of HIV do not mention strategies for dealing with PLWH who are mental health users, nor how to manage neuropsychiatric side effects caused by efavirenz therapy (National Consolidated Guidelines for PMTCT of HIV and the Management of HIV in Children, Adolescents and Adults 2014:72). The guidelines do not contraindicate the use of efavirenz in every PLWH who is a mental health user, but only those manifesting severe symptoms. ‘Severe symptoms’ is left open to interpretation. According to the package insert of efavirenz (Adco-Efavirenz^®^ 2007), it is not contraindicated in PLWH who are mental health users, but recommends that these patients be closely monitored. Participants expressed a desire that further elucidation concerning this special population be provided in the guidelines:

‘…especially side effects and there should perhaps be, even just an addendum, attached for further enquiry.’ (Interview 12, MO, female)

‘…HIV and HIV associated neurocognitive disorders I couldn’t rely on only national guidelines….’ (Interview 2, MO, female)

‘…basically it’s not a holistic approach….’ (Interview 4, PN, male)

‘…identifying the patients uh who are who are vulnerable to these uh uh side effects in the first place.’ (Interview 8, MO, male)

Further studies concerning the effects of efavirenz and mental illness in PLWH are necessary to provide the kind of information participants are requesting.

## Conclusions

Uncertainty regarding basic definitions can undermine an entire system. The inability of many participants to provide a clear definition of a neuropsychiatric side effect in medical language may indicate a lack of appreciation of the importance of this particular phenomenon.

Participants indicated mixed feelings regarding the use of efavirenz in PLWH who are mental health users. Many of the participants were uncertain as to whether efavirenz may be used, although clinicians in psychiatric hospitals indicated that they had not encountered PLWH who are mental health users on efavirenz who needed to be switched to alternative therapy, such as nevirapine or lopinavir/ritonavir, because of worsening of psychiatric symptoms or intolerable neuropsychiatric side effects. Two participants mentioned that the use of the FDC has reduced the incidence of neuropsychiatric side effects compared to efavirenz being used as a single agent. The reasons behind this have not been established.

The place of efavirenz in the treatment of PLWH who are mental health users needs to be more clearly defined. Even though efavirenz is no longer being used in developed countries such as the United States, it has a wide presence in Africa as part of the recommended first-line treatment of HIV. Participants in this study expressed the desire for more information regarding its side effects and use in special populations.

## Recommendations

Proper integration of mental health services into the wellness programme at primary healthcare facilities is critical in order to effectively screen for patients who had a history of, or who are currently suffering from, mental disorders and treat them appropriately.

Further studies focusing on the incidence of psychiatric relapse in these patients would provide valuable information. The National ART Guidelines of South Africa need to be expanded to include side effects caused by all agents and suggestions for management strategies. This will serve to further empower healthcare staff with knowledge and will translate to better patient care and outcomes.
